# Clustering of unhealthy behaviours and subsequent sickness absence among Finnish municipal employees

**DOI:** 10.1093/eurpub/ckac129.311

**Published:** 2022-10-25

**Authors:** J Salmela, J Lahti, N Kanerva, O Rahkonen, A Kouvonen, T Lallukka

**Affiliations:** 1 Department of Public Health, University of Helsinki, Helsinki, Finland; 2 Department of Food and Nutrition, University of Helsinki, Helsinki, Finland; 3 Faculty of Social Sciences, University of Helsinki, Helsinki, Finland; 4 Centre for Public Health, Queen’s University Belfast, Belfast, UK

## Abstract

**Background:**

Unhealthy behaviours are associated with increased sickness absence (SA), but few studies have explored these associations using person-oriented approach. We aimed to identify latent classes of unhealthy behaviours among female and male employees and examined their associations with subsequent SA.

**Methods:**

Health behaviours (leisure-time physical activity, sedentary behaviour, fruit and vegetable [F&V] consumption, sleep, binge drinking, and use of tobacco products) were derived from the Helsinki Health Study questionnaire survey, collected in 2017 among 19-39-year-old employees of the City of Helsinki, Finland. The questionnaire data were linked to employer's SA register. Latent class analysis was used to identify underlying profiles of unhealthy behaviours and negative binomial regression was used to examine their associations with subsequent SA (≤7 days, >7 days, and all lengths) among 3228 women and 771 men. The mean follow-up time was 2.1 years.

**Results:**

Among women, we identified 3 latent classes: 1) healthy behaviours (81% of women), 2) binge drinking and tobacco use (12%), and 3) inadequate F&V consumption and insufficient sleep (7%). Classes 2 and 3 showed increased rates for subsequent SA compared to class 1, regardless of the length of SA spells (age-adjusted rate ratios [RR] 1.37-1.42 and 1.35-1.64, respectively). Among men, we identified 3 latent classes: 1) healthy behaviours (51% of men), 2) binge drinking and tobacco use (19%), and 3) inadequate F&V consumption, binge drinking and tobacco use (30%). While classes 1 and 2 were not different in terms of subsequent SA, class 3 had increased rates of subsequent, particularly short-term SA (RR 1.24, 95% CI 1.03-1.48).

**Conclusions:**

Preventive actions should consider simultaneously several unhealthy behaviours while aiming to reduce employees’ SA. These actions might benefit from regarding potential gender differences in the clustering of unhealthy behaviours and their associations with SA.

**Key messages:**

• Preventive actions to reduce sickness absence should consider clustering of unhealthy behaviours among employees.

• Potential gender differences need to be regarded in these actions.

